# Computational Analysis and Experimental Validation of Gene Predictions in *Toxoplasma gondii*

**DOI:** 10.1371/journal.pone.0003899

**Published:** 2008-12-09

**Authors:** Joseph M. Dybas, Carlos J. Madrid-Aliste, Fa-Yun Che, Edward Nieves, Dmitry Rykunov, Ruth Hogue Angeletti, Louis M. Weiss, Kami Kim, Andras Fiser

**Affiliations:** 1 Biodefense Proteomics Research Center, Albert Einstein College of Medicine, Bronx, New York, United States of America; 2 Department of Systems and Computational Biology, Albert Einstein College of Medicine, Bronx, New York, United States of America; 3 Department of Biochemistry, Albert Einstein College of Medicine, Bronx, New York, United States of America; 4 Department of Pathology, Albert Einstein College of Medicine, Bronx, New York, United States of America; 5 Department of Developmental and Molecular Biology, Albert Einstein College of Medicine, Bronx, New York, United States of America; 6 Department of Medicine, Albert Einstein College of Medicine, Bronx, New York, United States of America; 7 Department of Microbiology and Immunology, Albert Einstein College of Medicine, Bronx, New York, United States of America; 8 Laboratory for Macromolecular Analysis and Proteomics, Albert Einstein College of Medicine, Bronx, New York, United States of America; University of Maryland, United States of America

## Abstract

**Background:**

*Toxoplasma gondii* is an obligate intracellular protozoan that infects 20 to 90% of the population. It can cause both acute and chronic infections, many of which are asymptomatic, and, in immunocompromized hosts, can cause fatal infection due to reactivation from an asymptomatic chronic infection. An essential step towards understanding molecular mechanisms controlling transitions between the various life stages and identifying candidate drug targets is to accurately characterize the *T. gondii* proteome.

**Methodology/Principal Findings:**

We have explored the proteome of *T. gondii* tachyzoites with high throughput proteomics experiments and by comparison to publicly available cDNA sequence data. Mass spectrometry analysis validated 2,477 gene coding regions with 6,438 possible alternative gene predictions; approximately one third of the *T. gondii* proteome. The proteomics survey identified 609 proteins that are unique to *Toxoplasma* as compared to any known species including other Apicomplexan. Computational analysis identified 787 cases of possible gene duplication events and located at least 6,089 gene coding regions. Commonly used gene prediction algorithms produce very disparate sets of protein sequences, with pairwise overlaps ranging from 1.4% to 12%. Through this experimental and computational exercise we benchmarked gene prediction methods and observed false negative rates of 31 to 43%.

**Conclusions/Significance:**

This study not only provides the largest proteomics exploration of the *T. gondii* proteome, but illustrates how high throughput proteomics experiments can elucidate correct gene structures in genomes.

## Introduction

*Toxoplasma gondii* is an obligate intracellular protozoan, belonging to the phylum Apicomplexa and is an important pathogen in both immune competent and immune compromised humans. The parasite causes chronic infection in adults and is present in an estimated 22.5% of people older than 12 in the United States [1] and up to 90% of the population in other regions of the world [2]. Acute infection is typically not symptomatic. Reactivation of latent infections is seen in immune compromised individuals, where infection often presents as encephalitis. *T. gondii* clinical disease is most typical in immune compromised individuals and is a common opportunistic pathogen associated with AIDS.

T. gondii has a wide range of hosts including almost all mammals as well as birds. It exists in three life stages. Oocysts are produced in the definitive host, the cat, and are environmentally resistant, surviving for prolonged periods of time in water, thus causing potential waterborne illness. Bradyzoites, found in the intermediate hosts, are slow growing parasites contained in vacuoles, which form tissue cysts and are generally unrecognized by the host's immune system. Bradyzoite tissue cysts can be transmitted via ingestion of undercooked, infected meat products or contaminated water. Once the parasite is present in the host, bradyzoites or sporozoites differentiate into the tachyzoite stage, which is responsible for the dissemination and clinically apparent infection. Due to waterborne outbreaks associated with ocular toxoplasmosis [3], *T. gondii* is classified by the National Institute of Allergy and Infectious Diseases as a Category B priority pathogen.

*T. gondii* is an important model system for the phylum Apicomplexa [4], which includes, among others, Plasmodium (malaria) and *Cryptosporidium* species. Unlike many other Apicomplexa, which are experimentally intractable, *T. gondii* is easily cultured in *vitro*, has well established experimental protocols for genetic manipulation, and has a well characterized mouse model [5].

A whole genome shotgun sequence of T. gondii has been generated by The Institute for Genomic Research (TIGR; now the J. Craig Venter Institute) at 12× coverage (available at http://www.toxodb.org), and additional genome sequence is available from the Sanger Centre (http://www.sanger.ac.uk/Projects/Protozoa). Initially, three gene prediction algorithms, TigrScan [6], TwinScan [7], and GlimmerHMM [6], were employed by TIGR and ToxoDB.org to identify genes in the ME49 strain of T. gondii. TigrScan and GlimmerHMM are Generalized Hidden Markov Model, *ab initio* gene prediction methods. The two models differ in their underlying statistical methods; TigrScan employs weight matrices and Markov chains, while GlimmerHMM incorporates additional splice site models. TwinScan combines comparative genomics and the probability model approach by integrating local genomic alignments from related species and *ab initio* methods. TwinScan was run using *Eimeria tenella* as the informant sequence. A fourth dataset of ME49-strain gene predictions was developed by ToxoDB and is currently available from version release 4.3 [8]. The Release4 predictions were produced using GLEAN [9], an algorithm that creates consensus gene predictions by integrating available experimental data such as expressed sequence tags (ESTs) and proteomics data. The computational gene prediction methods produce protein sequence sets that represent the theoretical proteome of *Toxoplasma gondii*; however, the datasets of predicted protein sequences are quite different and are likely to introduce substantial inaccuracy [10]–[12]. Without experimental verifications it is not possible to assess whether any of the datasets offer a comprehensive view of the *T. gondii* proteome or which datasets, if any, are more accurate than the others.

Large scale proteomics approaches have been used to analyze genomes of various organisms such as *S. cerevisiae* [13], *M. mobile* [14], *C. parvum* [15], *T. gondii* [16] and *S. luteogriseus* [17]. Targeted studies of *T. gondii* rhoptry [18], secretory [19], and micronemal [20] proteins highlight the value of applying proteomics to explore important subproteomes. Further, proteomics can be used to elucidate the role of post-translational modifications, such as N-glycosylation, in the function of important proteins [21].

We hypothesize that a systems-level analysis of the *T. gondii* proteome, using an approach that integrates proteomics and bioinformatics, will identify novel proteins that represent unique chemotherapeutic targets or have important biological functions during the obligate intracellular development of the parasite. We assembled a database comprising all computationally and experimentally derived sequences in an effort to capture the complete hypothetical proteome of *T. gondii*. Functional annotation of the proteome, using motif prediction methods, helped to gain insight into the biological relevance of the predicted proteins. Comparative genomics identified those proteins that were unique to *T. gondii*. Finally, MS and EST data were mapped to the experimentally derived and computationally generated proteins to experimentally validate predicted sequences and to assess the accuracy of various gene prediction methods.

## Results

### High throughput proteomics analysis of *T. gondii*

We explored the *T. gondii* proteome by performing tandem mass spectrometry (MS/MS) experiments on *T. gondii* plasma membrane, cytoskeletal and cytosolic protein preparations. A total of 252 MS/MS experiments provided 7,270 (203,990) unique (and redundant) proteolytic peptides with an average false discovery rate of approximately 2% based on peptide detection in a “decoy” database (Table 1). The decoy database was generated by Mascot (www.matrixscience.com) in the following way: during the search, every time a protein sequence from the target database is tested, a random sequence of the same length is automatically generated and tested. The average amino acid composition of the random sequences is the same as the average composition of the target database. Peptides were searched against human and mycoplasma protein databases to remove potential contaminating peptides. The MS/MS peptides were searched against the hypothetical *T. gondii* proteome, comprised of all computationally predicted and experimental sequences, to identify experimentally supported sequences. Expressed sequence tag data was used to provide further experimental support for some proteins in the *T. gondii* proteome. All MS data and analysis is publicly accessible in full depth at the Web site of the Albert Einstein Biodefense Proteomics Research Center (http://www.fiserlab.org/biodefense and http://www.fiserlab.org/epicdb ) and summaries have been deposited to the NIH maintained Resource Center for Biodefense Proteomics Research, website http://www.proteomicsresource.org/ and to ToxoDB (http://www.toxodb.org).

**Table 1:** Number, average length, and average MASCOT score for all peptides identified by MASCOT when the MS results were searched against the amino acid sequences in the combined dataset.

Number of MS/MS experiments	252
Number of identified MS/MS peptides	
redundant peptides	203,990
unique peptides	7,270
Average peptide length (residues)	15.3
Average MASCOT ion score	63.14

### Predicted and experimentally known proteins of *T. gondii*

The hypothetical proteome, comprised of 30,197 amino acid sequences, combines predicted proteins from the TigrScan (8,336 sequences), TwinScan (7,588), Glimmer (4,954), and Release4 (7,793) datasets, and the available *T. gondii* sequences from the NCBI non-redundant protein database (1,526) (NR).

**Figure 1:**
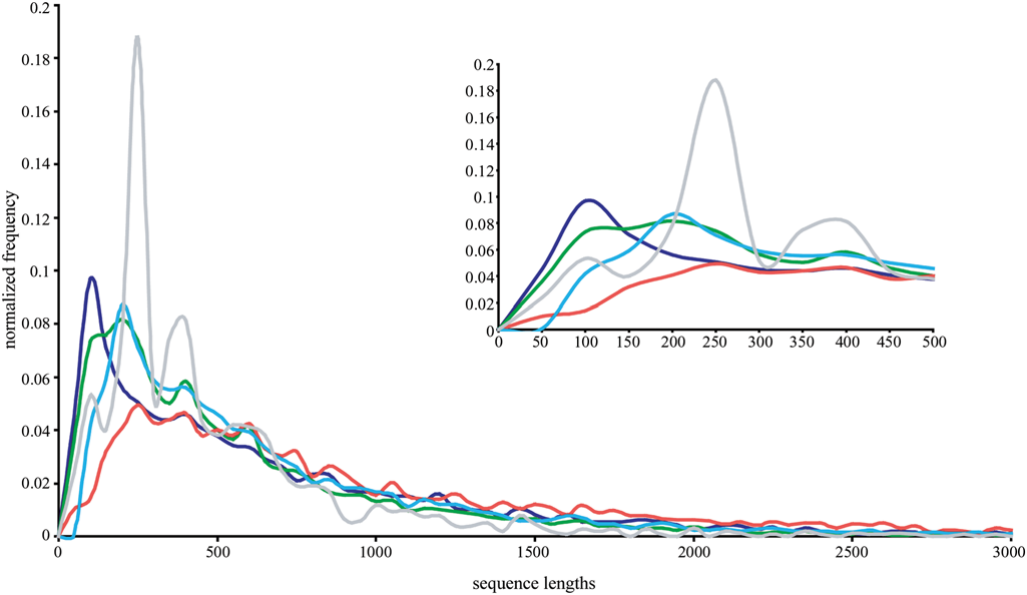
Frequency distribution of sequence lengths (amino acid residues per sequence) for TigrScan (dark blue), TwinScan (green), Glimmer (red), Release4 (light blue), and NR (grey) sequences. The frequencies are normalized to the total number of sequences in each dataset. The large graph shows the distributions for sequence lengths of 0–3,000 amino acids. The inset shows the same distributions but for sequence lengths of 0–500 amino acids.

The majority of the sequences in the combined dataset (94%) are less than 2,000 residues long though some sequences are as long as 14,514 residues. The averages, ranges, and distributions of the sequence lengths already suggest substantial differences among the protein datasets (Table 2, Fig. 1). Glimmer predicted sequences (1,077 residues average length) are, on average, much longer than the TigrScan, TwinScan, Release4, or NR sequences (681, 614, 719, and 510 residues average length, respectively). Furthermore, the maximum sequence length for TigrScan (9,696 residues) is substantially shorter than the maximum lengths of the TwinScan, Glimmer, Release4, or NR sequences (13,936, 14,514, 11,862, and 12,269 residues, respectively). The NR sequences have the shortest average length (510 residues) and the distribution shows the highest frequency of sequences of approximately 200–300 residues. The median lengths for each distribution, compared among each dataset, mimic the characteristics of the differences in average lengths. The Glimmer sequences had the highest median length (695), followed by Release4 (472), TigrScan (457), TwinScan (395), and the NR sequences (341). The shorter average and median lengths of the NR sequences is not necessarily an inherent characteristic of *T. gondii* proteins but could be an artifact because the researched proteins are likely not a representative sample of the *T. gondii* proteome. Rather, the predominance of shorter sequences could be a consequence of the greater ease of studying these proteins experimentally, especially in the past.

**Table 2:** Length characteristics (average, standard deviation, and range) and experimental data (MS peptides, MS coverage, EST alignments, MS *AND* EST data), for sequence of each prediction type.

	Predicted Protein Types				
	TigrScan	TwinScan	Glimmer	Release4	NR
**Total Number of Sequences**	8,336	7,588	4,954	7,793	1,526
**Length Characteristics**					
Sequence Length, average±stdev	681±733	614±743	1,077±1,168	719±803	510±676
Sequence Length, range	20–9,696	2–13,936	23–14,514	50–11,862	1–12,269
**Experimental Data**					
Sequences with Assigned MS peptides (% of dataset)	17%	19%	27%	20%	49%
Average sequence coverage of MS peptides	10.7%	13.4%	8.6%	12.5%	29.9%
Sequences with Filtered EST alignments (% of dataset)	58%	62%	75%	72%	85%
Sequences with MS peptides AND EST (% of dataset)	15%	17%	25%	18%	48%

The substantial differences in the length characteristics of the predicted proteins suggest that the algorithms either predict sequences from vastly different genes and coding regions or that there are significant disparities in the prediction of the structure and splicing of the exons and introns within similar gene coding regions.

### Comparing protein sequences obtained from different prediction methods

Identical or nearly identical proteins in the hypothetical proteome were grouped in order to explore the overlap and differences among the computational gene prediction methods. Each gene finder was employed on the same genome and, consequently, the four methods ideally should have predicted identical genes. Of course, conversely, the algorithms may have also predicted different splicing among predicted exons of the same open reading frame, predicted different open reading frame initiation or termination points, or added and/or missed entire open reading frames, thus generating some vastly different proteins. If each gene prediction method (TigrScan, TwinScan, Glimmer, and Release4) produced the same predicted sequence, those sequences would be clustered together. Grouping identical proteins in the combined dataset results in 25,908 clusters (86% of the original set), of which, only 101 contain predicted sequences from all four methods (Table 3). The numbers of identical sequence groups with a sequence from one (22,556 groups), two (2,058), three (521), or all four (101) prediction methods confirms the observation that the gene prediction methods produce very different results, with many unique sequences for each prediction method. The percentage of unique sequences that is produced by each prediction method is roughly similar for the TigrScan, TwinScan, and Glimmer methods (76–87% of all predictions), with Release4 having a slightly smaller percentage of unique sequences (68%) (Table 3). In general, any two prediction methods share less than 12% (and as low as 1.4%) identical predicted genes in a head-to-head comparison. The above analysis does not insinuate that any one gene finder algorithm is superior to the others. However, the results provide an initial basis for the effort of examining all available sequences from each computational annotation of the genome when examining the proteome.

**Table 3:** Number of identical sequence groups containing one, two, three, or all four types (TigrScan, TwinScan, Glimmer, Release4) of protein prediction methods.

Overlap Among Prediction Methods	Number Of Identical Sequence Groups
All (4) Prediction Methods Agree (Tigr,Twin,Glimmer,Release4)	101
Three Prediction Methods Agree	521
Two Prediction Methods Agree	2,058
Single Prediction Method	22,556
	unique Tigr = 7,245
	unique Twin = 5,778
	unique Glimmer = 4,252
	unique Release4 = 5,281

### Clustering predicted proteins by sequence similarity

**Figure 2:**
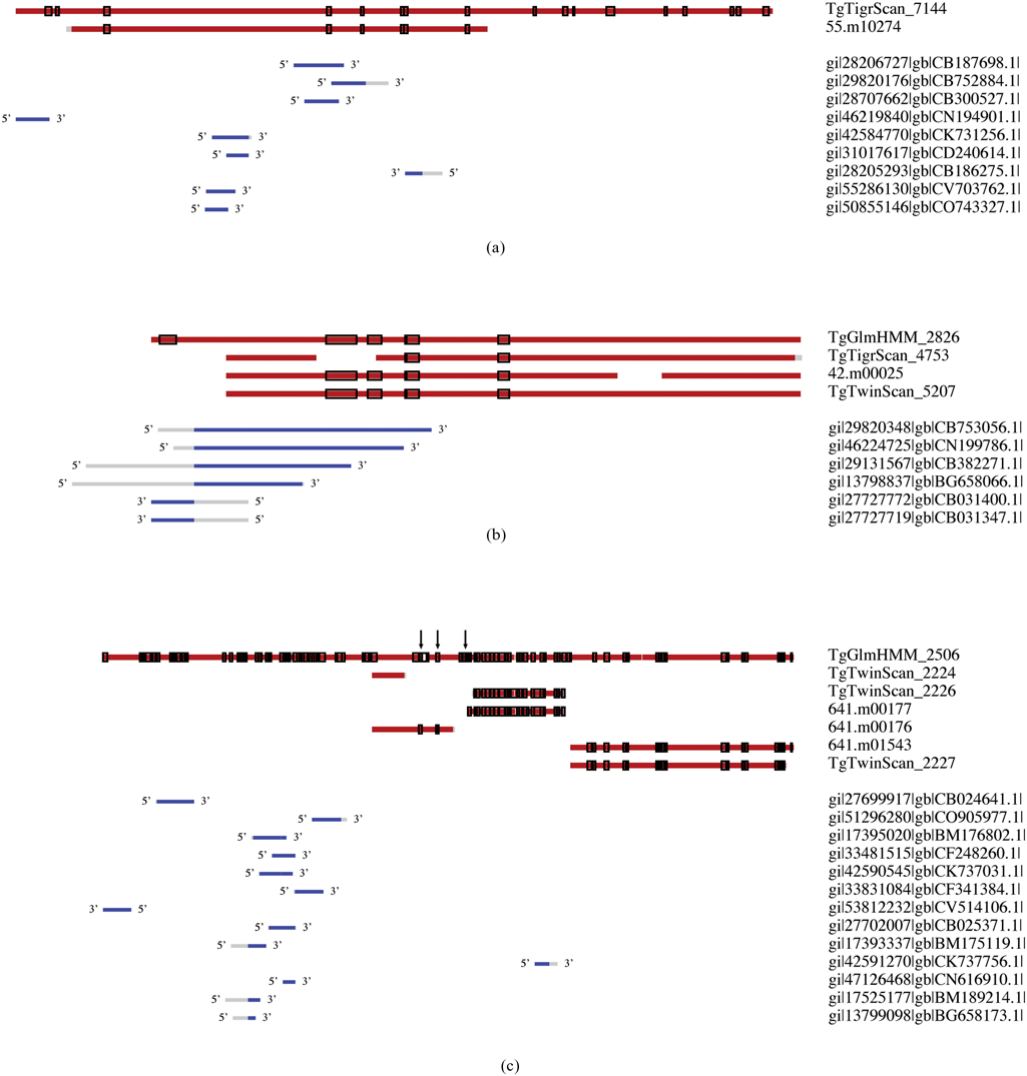
Examples of clusters of similar predicted amino acid sequences (red lines) with MS peptide hits (shown as open black blocks on amino acid sequences) and aligned ESTs (blue lines below amino acid sequence clusters), with EST translation directionality indicated. Grey lines indicate areas where sequences differ and are not aligned. Identifiers are listed on the right. a. Cluster of a TigrScan (TgTigrScan_7144) and Release4 (55.m10274) sequence. The TigrScan sequence has additional 5′ and 3′ regions (exons) compared to the Release4 sequence. The additional regions are verified by MS hits. There are no TwinScan, Glimmer, or known NR sequences in this MS validated coding region. b. Cluster of a Glimmer, TigrScan , Release4, and TwinScan sequence, all validated by MS peptide hits. The Glimmer sequence (TgGlmHmm_2826) has an additional MS validated 5′ region. The TigrScan ( TgTigrScan_4753) sequence does not have an exon that is included in the Glimmer, Release4, and TwinScan sequences and that is supported by both MS and EST data. c. Cluster of a Glimmer (TgGlmHmm_2506) sequence, three TwinScan (TgTwinScan) sequences, and three Release4 sequences. The TwinScan and Release4 methods each predict three distinct proteins from this coding region whereas the Glimmer method predicts one. The distribution and location of the MS peptides on the Glimmer sequence indicates that the full length protein is likely to be present in the proteome. However, the shorter splice variants may also be real proteins. An example of the proteomics data confirming the existence of a continuous portion of the sequence is the group of MS peptides indicated by arrows.

Discrepancies in protein sequences that are transcribed from similar genomic locations, whether the result of prediction differences or splicing variability, likely account for most of the disparities among the protein datasets. Therefore, in order to assess the fraction of sequences that are not identical yet share a common part and, thus, may have been derived from the same genomic location, the hypothetical proteome of 30,197 sequences was clustered with a 90% sequence identity threshold requirement. The clusters were constructed by aligning each sequence with the longest sequence in a cluster, which is called the “representative sequence” (see Methods), while allowing large gaps in the alignment. The number of identical amino acid residues within regions of sequence overlap was normalized over the length of the shorter sequence to determine the sequence identity between the aligned sequences. This approach can group together sequences with very different lengths if the overlapping parts share more than 90% sequence identity. This clustering strategy is an obvious way to assess redundancy among the datasets, but it is also a way to examine potential splicing variability within the genome. Alternative splicing events such as alternative 5′ or 3′ exon splice sites, skipped exons, or retained introns result in sequences that are splice variants of the same gene. Although the sequences are different, the local similarities allow them to be clustered together (Fig. 2a–c).

Clustering by sequence similarity allows for an interpretation of a comprehensive set of genes and open reading frames that are predicted by various gene finder algorithms. A cluster of similar sequences (or a single sequence) is representative of a predicted gene at the genomic locus despite the fact that there may be some discrepancy in the exact structure of the gene in question due to differences in the predicted sequences. This method of clustering by local sequence similarity is a distinct analysis compared to the evaluation of identical sequence overlap produced by the gene finder algorithms. Additionally, the local sequence-similarity clustering, which encompasses all subsequent clustering analysis, was performed on the redundant set of 30,197 protein sequences because the ensuing statistics regarding the differences among the prediction methods would be artificially affected if some sequences were removed from the dataset in cases in which two or more prediction algorithms produced identical sequences.

The hypothetical proteome of 30,197 sequences collapses to 14,983 non-redundant clusters with an average size of 2.02 sequences. The majority of the clusters (55%) are individual sequences (Table 4), strengthening the observation that there are many unique sequences that do not share even a common sub-sequence with other proteins and that the predicted protein datasets provide remarkably different alternatives. If the 8,281 unique sequences (singleton clusters) are removed from the original combined dataset, there are 21,916 remaining sequences that are clustered with at least one other sequence *i.e.*, about 73% of the combined dataset is redundant, based on sequence similarity. The elimination of the singleton clusters was performed only for the aforementioned evaluation of the degree of redundancy in the dataset. The singleton clusters were included in all other analyses.

**Table 4:** Distribution of cluster sizes (number of sequences per cluster) for the CD-HIT clustered combined protein dataset.

Cluster Size (sequences per cluster)	Number of Clusters
1	8,281
2	2,476
3	1,850
4	1,577
5	430
6	160
7	104
8	42
9	23
10	12
>10	28

The percentages of the clusters that contain a TigrScan, TwinScan or Release4 sequence are similar (53%, 47%, and 49%, respectively), while the percentages that contain a Glimmer or NR sequence are much lower (33% and 6% respectively) (Table 5). The differences for the Glimmer predictions and the NR sequences could be explained by the fact that these datasets, especially the NR, are significantly smaller then the datasets for the other three prediction methods. The small fractions of common protein predictions suggest that any single prediction dataset either covers a small portion of the theoretical *T. gondii* genome (∼50% or less) and/or produces a very large percentage of false positives.

**Table 5:** Number of all sequence clusters and clusters supported by MS data, EST data, and MS and EST data, respectively.

	Predicted Protein Types				
	TigrScan	TwinScan	Glimmer	Release4	NR
	**14,983 Total Clusters**				
sequence type (# clusters)	7,914	7,093	4,916	7,389	889
% of Total Clusters	53%	47%	33%	49%	6%
	**2,477 MS Clusters**				
sequence type (# clusters)	1,505	1,625	1,405	1,675	384
% of MS Clusters	61%	66%	57%	68%	16%
	**9,242 EST Clusters**				
sequence type (# clusters)	4,954	4,851	3,816	5,645	748
% of EST Clusters	54%	52%	41%	61%	8%
	**2,275 MS AND EST Clusters**				
sequence type (# clusters)	1,396	1,507	1,315	1,562	370
% of both MS and EST Clusters	61%	66%	58%	69%	16%

For each category, the number of clusters with each predicted sequence type is shown along with the corresponding percentage of the clusters in the respective category.

### Clustering by genomic location

Clustering proteins by sequence similarity may group together paralogs from different genomic locations that emerged through gene duplication or gene shuffling mechanisms. Therefore, we explored the homogeneity of the sequence clusters in terms of genomic localization. First, individual protein sequences were mapped onto the *T. gondii* genome. We successfully mapped 27,777 sequences (92% of the combined and redundant dataset) onto the genome (see Methods). The sequences span an average length of 5,879 nucleotides and, on average, approximately 52% of the genomic coding region is exonic. We found 787 sequences (2.6%) only that can be mapped to multiple locations in the genome, which suggests a relatively low level of gene duplication events.

Genomic mappings of the protein sequences determined the genomic location of 92% of the 14,983 sequence clusters (see Methods). In approximately 1% of the clusters, a sequence maps to a different genomic location than the rest of the cluster members. Approximately 99% of the clusters are composed of sequences that are transcribed from the same genomic regions. These clusters of predicted protein sequences may also represent possible splice variants of the same gene. There are 3,120 and 2,969 genomic regions that are comprised of either an individual cluster or a co-localized cluster group (see Methods), respectively, and thus, we estimate that there are 6,089 potential protein-coding regions in the *T. gondii* genome. The genomic protein-coding regions span, on average, 9,924 nucleotides and, since the *T. gondii* genome is about 65 Mb, approximately 7% of the genomic sequence is intergenic. The estimated percentage of the genome that accounts for coding regions is larger than what has been previously reported for chromosome Ia (56.7%) and Ib (58.1%) [25], which could be a reflection of the increased number of false positive protein sequences that arise from a larger variety of analyzed gene prediction algorithms or possible characteristic differences among the *T. gondii* chromosomes.

### High throughput experimental validation of the *T. gondii* proteome

We explored the *T. gondii* proteome by performing high throughput, tandem mass spectrometry (MS/MS) experiments on *T. gondii* plasma membrane, cytoskeletal, and cytosolic protein preparations and cross referencing the MS-derived peptides with the computationally generated and experimentally derived amino acid sequences of the hypothetical *T. gondii* proteome. Expressed sequence tag data was used to provide further experimental support for some sequences in the hypothetical *T. gondii* proteome.

The 252 MS/MS experiments provided 7,270 unique proteolytic peptides that identified 6,438 sequences (21% of the combined dataset). The average peptide coverage of the MS/MS supported sequences is 13.5%. The percentages of sequences with an assigned peptide are similar for the TigrScan, TwinScan, Glimmer, and Release4 datasets (17%, 19%, 27%, and 20%, respectively) while the percentage is substantially higher for the NR dataset (49%) (Table 2). The average peptide coverages are also similar for the TigrScan, TwinScan, Glimmer, and Release4 sequences yet significantly higher for the NR sequences (10.7%, 13.4%, 8.6%, 12.5%, respectively, compared to 29.9%) (Table 2). The fact that the NR dataset has a higher percentage of sequences with assigned peptides and a higher average coverage seems intuitive as is a reflection of the fact that these sequences are believed to be more reliable, because, in general, they are experimentally derived. These sequences certainly do not represent a comprehensive or uniform sample of the *T. gondii* proteome and thus, no assumptions can be made about the quality of the sequences or coverage of the proteome by this dataset with respect to the predicted datasets. The observation that each protein prediction method has a similar degree of experimental validation, along with the disparate nature of the sequences predicted by each method, indicates that each method predicts the proteome with a similar level of accuracy.

There are 2,477 clusters (17% of the total clusters) that contain a sequence with an assigned MS/MS peptide. The portions of the MS/MS supported clusters that contain a TigrScan, TwinScan, or Release4 sequence are similar (60.75%, 65.60%, and 67.62%, respectively) while the portion that contains a Glimmer sequence is slightly lower (56.72%) (Table 5). The discrepancy in the accuracy of the protein prediction methods can be explained by the fact that there are fewer Glimmer sequences as compared to the other prediction methods. These data show that none of the full genome predictions manages to identify all of the proteins that are supported by MS/MS data; each prediction method is missing 31 to 43% of the validated part of the proteome. In these calculations we assumed that if there is at least one proteolytic peptide that matches a sequence in a cluster, then all sequences are validated. However a proteolytic peptide may not have an overlap with all sequences in a cluster (Fig. 2a–c). Nevertheless for each type of protein, due to the relatively dense coverage of MS peptides in each cluster, a sequence that was present within an MS/MS supported cluster almost always had a peptide mapped directly to the sequence (≥90% of the occurrences of a sequence within the MS/MS supported cluster). This indicates that there is experimental evidence to support each sequence within the cluster, despite differences in splicing and/or predictions among the clustered sequences.

According to the various prediction algorithms, the *T. gondii* genome is expected to encode approximately 7,800 genes [26], [27], of which 18% were estimated to be life stage specific (∼1,400) [28]. This suggests that our high throughput MS/MS experiments explored at least one third of the genome of *T. gondii* tachyzoites (2,477 clusters). This may be an underestimate if a significant fraction of our clusters represent two or more gene products.

EST mapping analysis of the hypothetical *T. gondii* proteome offers similar conclusions to what were generated from the MS/MS analysis. It was possible to validate 20,123 sequences (67% of the combined dataset) with an aligned EST sequence, which corresponds to 9,242 clusters (62% of the total clusters). The TigrScan and TwinScan datasets have roughly the same percentages of sequences that are supported with an EST alignment (58% and 62%, respectively) while the Glimmer and Release4 methods have similar and higher percentages of sequences with an EST alignment (75% and 72%, respectively). The enrichment of Glimmer sequences with an EST alignment is likely a result of the Glimmer sequences being, on average, much longer then the TigrScan, TwinScan, or NR sequences. Thus, there is a greater chance that a Glimmer sequence will cover part of the genome that is also sequenced by an EST. The high percentage of Release4 sequences with an EST alignment is possibly the result of ESTs (or other genomic data) being included in the integrated data that was used to derive the Release4 sequences (Table 2).

The percentages of the EST supported clusters that contain TigrScan, TwinScan, or Release4 sequences are similar (54%, 52%, and 61%, respectively) whereas the percentage that contains a Glimmer sequence is lower (41%) (Table 5). A vast majority (≥96%) of the sequences within the EST supported clusters have an EST alignment, again indicating that each sequence, within the cluster, has experimental support despite possible splicing or prediction differences.

The EST alignments were filtered based on requirements for minimum alignment length and sequence identity in order to differentiate cases in which the amino acid sequences were supported by a strong alignment from the cases in which the alignment indicated a paralog rather than a direct validation of the sequence by genomic data. Without filtering, there are 21,989 sequences that have EST alignments, which is only 9.3% more than the number of sequences with filtered EST alignments (20,123 sequences) and which represents only a 6% increase in the percentage of the hypothetical proteome that is supported by experimental EST evidence. The consistency in the number of amino acid sequences with filtered and non-filtered EST alignments indicates that, within the subset of sequences that are supported by EST data, there are strong similarities between the protein sequences and the EST sequences.

The MS proteomics data were cross-referenced with the EST genomics data. There are 5,881 sequences (19.5% of the combined dataset) that are experimentally supported by both an assigned MS/MS peptide and a filtered EST alignment, which one might consider to be the most highly validated proteins in the compiled proteome. The sequences with both types of experimental data correspond to 2,275 clusters (15.2% of the total clusters). The average MS/MS peptide coverage of sequences that are supported by MS and EST data or exclusively by MS data alone is 14.3% and 5.25%, respectively. The difference in peptide coverage may be a result of the proteins with EST data being more highly expressed in the *T. gondii* proteome compared to those without EST alignments.

Clusters that are supported by both MS data and EST data have a comparable portion of Glimmer, TigrScan, TwinScan, and Release4 sequences (58%, 61%, 66%, and 69%, respectively) (Table 5). Therefore, the gene prediction algorithms exhibit a false negative rate of 31 to 42%, which is the portion of the experimentally validated coding regions (clusters) (MS and EST) that are completely missed by individual prediction methods. While it is not possible to precisely identify the false positive predictions (predicted amino acid sequences that are not real proteins) from these data, it is clear that the rate of false positive predictions must be substantial for each prediction method.

Figures 2a–c illustrate some of the sequence clusters with their corresponding experimental data. The sequence cluster in the first example (Fig. 2a.) is validated with MS peptide hits as well as EST data, but there are only two predicted protein sequences, one from TigrScan (TgTigrScan_7144) and one from Relase4 (55.m10274), i.e. all other prediction methods failed to predict the protein coding region that is represented by this cluster. Moreover, the peptide coverage illustrates that the longer TigrScan prediction is either a valid splice variant of the coded protein or the Release4 prediction failed to identify both the N and C terminal segments of the protein, both of which have been validated by MS data and, in the case of the N-terminal, EST data. The second example cluster (Fig. 2b.) is composed of sequences from all four prediction methods, albeit with significant differences among the sequences. For instance, the TigrScan prediction suggests two separate proteins in this clusters, or genomic location, while MS peptides and EST evidence points to a continuous protein sequence. In addition, the N terminal segment that is unique to the Glimmer prediction (TgGlmHmm_2826) is confirmed by both an MS peptide and two EST alignments. The third example (Fig. 2c.) shows a cluster with a variety of vastly different predictions from three methods (TigrScan fails to predict a protein in this cluster). The arrows pointing to the Glimmer prediction indicate MS peptides that confirm areas of the protein sequence where other models predicted gaps (TgTwinScan_2224 and TgTwinScan_2226 as well as 641.m00177 and 641.m00176), suggesting that the continuous protein sequence exists at least as one splice variant. In addition, the N-terminal tail of the Glimmer prediction is confirmed with a very high coverage of MS peptides and numerous EST alignments, while this segment was missed by all other prediction methods.

### Functional annotation of the *T. gondii* proteome

Each protein sequence in the combined dataset was scanned against the PFAM database [31], a collection of protein domains and families, using pfam_scan.pl version 0.5, available from ftp.sanger.ac.uk. The output was parsed to find the PFAM domain names and locations for each sequence. Phobius (version 1.01) [32], a combined transmembrane topology and signal peptide predictor, was used to perform the transmembrane domain and signal peptide predictions.

There are 13,430 sequences (45% of the combined dataset) with at least one PFAM domain annotation compared to 20,326 sequences (67%) that are annotated with orthologs predicted from pairwise BLAST [22] alignments with sequences from the NCBI NR database. The percentage of proteins that have PFAM domains compares well with the published results of PFAM 22.0, which reports that 49% of 213 *T. gondii* UniProtKB/TrEMBL deposited proteins have at least one PFAM domain [31]. Sequences with PFAM domains correspond to 5,741 clusters (38% of the total clusters). The TigrScan, TwinScan, and Release4 sequence sets have similar percentages of sequences with PFAM domains (36%, 43%, and 44%, respectively). The percentages of sequences with PFAM domains are similar for the Glimmer and NR sequence sets (62% and 69%, respectively). The higher proportion of PFAM domains found in the NR sequences, compared to TigrScan, TwinScan, or Release4, is probably a consequence of a higher level of confidence in these sequences, as they are typically experimentally derived. However, the higher proportion of PFAM domains for the Glimmer sequences is likely a consequence of the fact that they are much longer, on average, than the other sequence types (Table S1), so an individual sequence will coincide with a bona fide coding region within the genome with a higher probability than the other protein types. In summary, PFAM annotations are used to infer a certain degree of biological validity for approximately 45% of the compiled *T. gondii* proteome.

There are 6,927 sequences (23% of the combined dataset) that have predicted transmembrane domains, which corresponds to 3,730 clusters (25% of the total clusters), and which is consistent with the proportion of membrane proteins that have been predicted in other genomes (20–35%) [33]. There are 4,330 sequences (14% of the combined dataset) that have predicted signal peptides, which corresponds to 2,601 clusters (17% of the total clusters). The percentages of sequences with signal peptides and transmembrane regions are consistent among the individual datasets. Transmembrane proteins of *T. gondii* are of particular interest because of their role in the interaction and adhesion of the parasite to the host cell and their link to the virulence of the parasite. These critical functions make some subsets of membrane proteins good candidates for chemotherapeutic targets. Meanwhile sequences with signal peptides generally refer to proteins that are secreted from the cell. In the case of *T. gondii*, secreted proteins include those of the micronemes, rhoptries, and dense granules, which are integral parts of the unique process of interaction, invasion, and infection of the host cell by *T. gondii* [34].

### Comparative genomics of the *T. gondii* proteome

Each sequence was compared against other Apicomplexan genomes, against the human genome, and against the complete NCBI NR database (Table S1).

The amino acid sequences of the hypothetical proteome of *T. gondii* were searched against the complete NCBI NR database, Apicomplexa proteins, and human proteins to identify unique and conserved proteins. In general, 67% of the hypothetical *T .gondii* proteome (59% of the clusters) has a homologous sequence in NR and 64% has an Apicomplexan ortholog (58% of the clusters). Approximately 52% of *T. gondii* sequences (57.5% of the clusters) are unique as compared to the human genome and, thus, fulfill a primary requirement of chemotherapeutic target candidates.

### EPIC-DB: Experimental ProteomICs DataBase

All experimental data, annotations, protein cluster information and comparative genomics data are organized into a relational database (EPICDB) that is publicly accessible at http://www.fiserlab.org/epicdb. In addition, a summary of the data and information about reagents generated from the *Toxoplasma gondii* MS experiments are available from the Resource Center for Biodefense Proteomics Research website http://www.proteomicsresource.org/.

## Discussion

The proteomics study presented here is one of the largest of a genome and certainly the most comprehensive analysis and validation of the *T. gondii* proteome to date identifying novel proteins and offering unique insights into its gene structure. We experimentally validated with MS experiments 6,438 distinct proteins that can be clustered into 2,477 groups and account for approximately one third of the *T. gondii* genome.

Further, this analysis provides important novel information, with respect to the *T. gondii* proteome, since 3,838 (60%) of the experimentally identified proteins have been annotated as “hypothetical”, “putative” or “predicted” in the NCBI NR database. Additionally 609 MS identified *T. gondii* proteins are unique as compared to any known organisms, providing an important subset of validated proteins for drug targeting.

While the genomes of many organisms have been sequenced, there are relatively limited amounts of experimental protein data to accurately annotate these genomes or to validate the computational gene prediction methods. The current study offers a way to address this ubiquitous problem by integrating genomics and proteomics data. The large scale experimental validation of the hypothetical *T. gondii* genome shows a false negative rate for various gene prediction methods of about 31–42% (proteins that were confirmed experimentally by both MS and EST data but missed by one or more of the computational gene prediction methods) and experimentally confirms earlier anecdotal reports that gene prediction algorithms operate with a substantial false negative rate [29], [30].

This study demonstrates that genome analysis coupled with experimental proteomics data is essential to benchmark and improve the accuracy of gene prediction methods and facilitates insights into gene structure and splice variability within genomes.

## Materials and Methods

### Protein sequence datasets

All available *T. gondii* protein sequences were compiled from five datasets; TigrScan (8,336 sequences), TwinScan (7,588), GlimmerHMM (4,954), Release4 (7,793), and NR sequences (1,526). TigrScan, TwinScan and GlimmerHMM sequences were downloaded as FASTA files of protein sequences from http://www.toxodb.org/common/downloads/release-3.3/Genome/pep/. A FASTA file of Release4 sequences was downloaded from http://www.toxodb.org/common/downloads/release-4.0/TgondiiAnnotatedProteins.fa. The NR sequence dataset was obtained from the NCBI Entrez Protein Database, which was filtered, by the organism name, for *Toxoplasma gondii* (As of July 2008, predicted genes/proteins from *T. gondii* genome analysis have not been deposited to NCBI or GenBank databases). The five datasets were combined to provide a comprehensive set of all available protein sequences for the *Toxoplasma gondii* proteome and comprised 30,197 sequences. This is a redundant database as different prediction methods may identify the same sequence with different names.

### Identifying groups of nearly identical predicted protein sequences

Identical or nearly identical sequences in the combined dataset were grouped in order to assess the overlap among the four gene prediction methods. A BLAST [22] search was performed (without low complexity filtering and with an e-value of e<0.001) for each sequence against the combined dataset. Sequences in each alignment were evaluated for overlap and sequence identity. For sequences longer than 100 residues, two sequences in an alignment were considered identical if the sequential overlap and the sequence identity within the overlap were both 99% or greater. If both the query and subject hit sequences 100 amino acids or smaller the two sequences were required to be within one amino acid in terms of length and sequence identity. Groups containing only NR sequences (672 groups) were not included in the analysis of the computational prediction methods.

### Clustering the protein dataset

The combined, redundant dataset was clustered using CD-HIT [23]. The sequences are grouped by a given sequence identity threshold, which corresponds to the local sequence similarity. The longest sequence in each cluster is designated as the “representative” sequence, which is a CD-HIT terminology. The sequence identities are calculated for the shorter sequences with respect to the representative. The requirement to compare each sequence to the longest sequence in the cluster is inherent in the clustering algorithm. However, we do not extend this terminology to designate the longest sequence to literally represent all the clustered sequences. The band-width allowance for the sequence alignments was set to 1,000 gaps in order to compare sequences with very different lengths but that may share a short common segment. Clustering was performed in the most accurate mode where each sequence is added to the cluster to which it is the most similar rather than to the first cluster for which the sequence identity threshold is met. CD-HIT implements a greedy incremental clustering algorithm, which orders the sequences by decreasing length and makes the longest sequence the representative sequence of the first cluster. The next sequence is evaluated by being compared to the representative sequence of the original cluster and if it satisfies the sequence identity threshold it is added to the cluster. Otherwise it becomes the representative sequence of a new (second) cluster. Each successive sequence is evaluated by being compared to the representative sequences of each cluster and is either added to a cluster or becomes the representative sequence of a new cluster. One implementation of the algorithm clusters the sequences into the first cluster for which the minimum sequence identity threshold is satisfied. Thus, the first clusters could be artificially large. However, the clustering presented here evaluates each sequence against all the clusters and includes the sequence in the cluster for which it shows the highest sequence identity.

In order to evaluate the redundancy in the dataset, the sequences were clustered for decreasing sequence identity thresholds and the resulting numbers of clusters were analyzed. A linear relationship exists between the number of clusters and the sequence identity threshold for sequence identity threshold levels from 95% to 70% (data not shown). There is not a specific level of redundancy in the dataset that dictates inherent clusters of the sequences.

**Figure 3:**
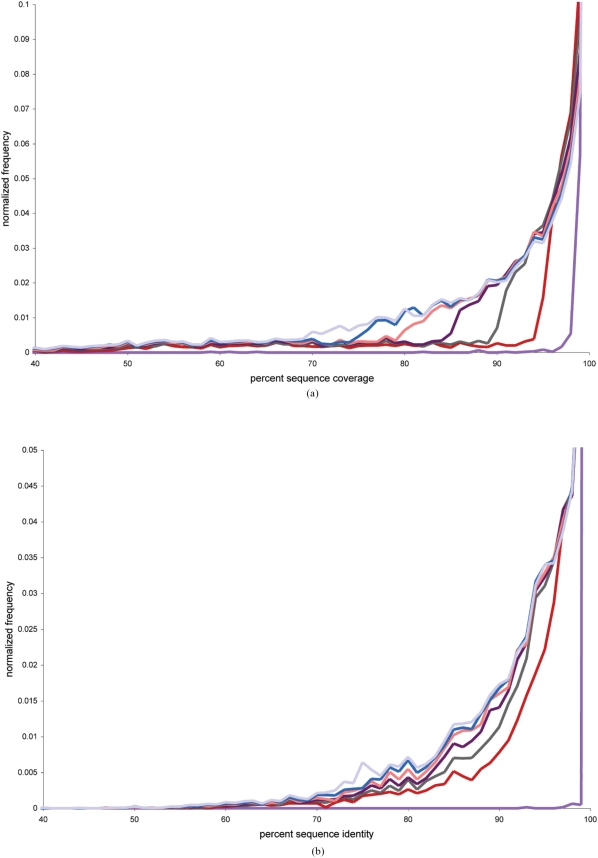
**a.** Histogram of distribution of pairwise sequence coverages within gene prediction clusters. Different curves refer to different sequence identity thresholds used for clustering. Color purple, red, brown, dark purple, pink, blue, cyan corresponds to cutoff values of 100%, 95%, 90%, 85%, 80%, 75%, 70%, respectively. **b.** Histogram of distribution of pairwise sequence identities within gene prediction clusters. Different curves refer to different sequence identity thresholds used for clustering. Color purple, red, brown, dark purple, pink, blue, cyan corresponds to cutoff values of 100%, 95%, 90%, 85%, 80%, 75%, 70%, respectively.

For each cluster within a CD-HIT sequence identity threshold level, the representative sequence was aligned to all other sequences in the cluster using BLAST2SEQ (without gaps and without low complexity filtering). The BLAST sequence identity and alignment coverage were evaluated for each sequence alignment. The sequence identity was defined as the number of identities in the alignment divided by the alignment length. The alignment coverage was defined as the alignment length divided by the length of the shorter sequence. The frequency of intra-cluster BLAST alignment percent identities and alignment coverages were computed for various CD-HIT sequence identity threshold levels from 100% to 70%. As the CD-HIT sequence identity threshold level decreases the frequency of cluster members with lower quality alignments increases. However a distinct shoulder forms around the 90–95% sequence identity clustering level, therefore we identified the 90% sequence identity threshold as the lower limit of sequence identity that produces clusters of highly homologous proteins (Fig. 3a–b).

### Mapping protein sequences to genomic locations

The genomic sequence for the ME49 strain was downloaded from ToxoDB.org. Each chromosome's nucleotide sequence was translated in 3 consecutive reading frames in both the forward and reverse directions. A dataset of 2,622,472 potential open reading frames was created by dividing the 6-frame translations into any sequences that occurred between two genomic STOP codons. To determine the genomic location of a protein sequence a BLASTP (without low complexity filtering and without gaps) search was performed for the protein sequence against a custom database of the 2,622,472 generated open reading frames. The resulting local alignments were filtered to eliminate any alignments with an e-value greater than 10^−4^ or a percent identity below 80%. For cases in which the protein sequence was spliced to multiple exons in the genome the BLAST search produced multiple, highly significant, local alignments rather than aligning the complete protein sequence to a single genomic interval. Our mapping algorithm examined all possible combinations of local alignments in order to identify cases in which a sequence could have multiple mapping options to the genome, *i.e.*, duplicated genes. Each valid alignment configuration was converted to genomic nucleotide positions since the chromosomal locations of the open reading frames were known. This process was repeated for each local alignment within the protein query thus elucidating the physical mapping as well as the intron/exon structure of the protein sequence. 80% of the sequence was required to be mapped to the genome in order to constitute a successful mapping of the protein to the genome.

There are 2,420 sequences, of the 30,197 sequences in the combined dataset, that can not be mapped to the genome; 155 sequences have no BLAST alignments to any of the translated genomic open reading frames, 376 sequences have no BLAST alignments that meet the significance requirements, and 1,889 proteins are not mapped because significant portions of the sequences cannot be aligned to the genome. The unmapped sequences are likely to be predicted from scaffolds that are not integrated into the genome.

### Genomic locations of sequence clusters

The genomic locations of the protein sequences were used to localize CD-HIT clusters. The location of a genomic region of a CD-HIT cluster was defined by the sequence within the cluster that spanned the longest genomic region, including exons and introns. The genomic locations of each sequence, within a cluster, were compared in order to identify cases when proteins with similar sequences were derived from different genomic locations. The genomic locations of the sequence clusters were used to determine the distribution of the coding regions in the genome. Each chromosome was scanned in both directions. Clusters that had overlapping genomic regions and the same translation directions were grouped into co-localized cluster regions. The genomic regions spanned by the co-localized cluster regions were defined as potential protein coding regions.

### Growth of *T. gondii in vitro*

RH strain *T. gondii* were grown in human fibroblasts in DME media with 10% fetal calf serum and 1% Penicillin Streptomycin (GIBCO-BRL) in a 5% CO_2_ incubator. Parasites were transferred to fresh culture human fibroblast monolayers biweekly. The absence of Mycoplasma contamination was monitored monthly using a PCR method (GIBCO-BRL).

### Purification of membrane and cytoskeleton protein from *T. gondii*

1.2×10^10^ purified *T. gondii* RH strain tachyzoites from tissue culture were resuspended in 20 ml of SMDI buffer (250 mM Sucrose, 10 mM MOPS-KOH, pH 7.2, 2 mM DTT, 1× protease inhibitor cocktail) and disrupted by French press at a pressure of 1000 PSI, medium setting. The lysate was centrifuged at 756×g at 4°C for 10 min to pellet unbroken cells. Intact parasites and large debris were resuspended in 10 ml SMDI buffer and disrupted once more by French press at a pressure of 1000 PSI, medium setting. The pooled supernatant was centrifuged at 25,000×g at 4°C for 20 min. The supernatant was saved for analysis as the cytosolic fraction. The pellet was resuspended in 10 ml of 30% Percoll in SMDI buffer. After centrifugation at 75,000×g in an ultracentrifuge (Rotor TLA 100.3; 30,000 rpm) at 4°C for 25 min, the top band was collected from the self-generated gradient. The band was diluted in SMDI buffer and spun at 100,000×g for 90 min. at 4°C (Rotor TLA 100.3; 40,000 rpm). A band collected between the buffer and resultant Percoll cushion contained the *T. gondii* ghosts consisting of membranes and cytoskeleton [18].

#### Membrane fraction

To isolate the membrane fraction the *T. gondii* ghosts were resuspended in an equal volume of 2% thioglucopyranoside in 40 mM Tris pH 7.6 by pipeting the mixture up and down 10 times (suspension kept on ice) followed by a brief vortex. After centrifugation at 20,000×g in an Eppendorff centrifuge for 20 min. at 4°C, the supernatant was saved as membrane fraction (extraction 1). This extraction was repeated twice with 300 µl of 1% thioglucopyranoside (to make extractions 2 and 3). These fractions were then frozen in liquid nitrogen and stored at −80°C until used for protein analysis.

#### Cytoskeleton fraction

Insoluble material, which remained after the membrane fraction extraction, was washed twice with 40 mM Tris pH 7.6. Following this washing step the material was solubilized in 500 µl of urea lysis buffer (7.5 M Urea, 2.5 M Thiourea, 40 mM Tris pH 7.6, 2.5% Octyl-b-glucoside, 6.25 mM TCEP, 1.25× Proteinase inhibitor) followed by homogenization on ice 10 times using a Potter homogenizer. The material was then centrifuged at 8,000 rpm for 10 min at 4°C and the supernatant was collected and saved. This extraction was repeated twice on the remaining pellet using 300 ul of the urea lysis buffer and the supernatants were pooled with the initial extraction to produce the cytoskeleton fraction. This fraction was then frozen in liquid nitrogen and stored at −80°C until used for protein analysis.

### High throughput mass spectrometry

Nanospray LC-MS/MS was performed on a LTQ linear ion trap mass spectrometer (LTQ, Thermo, San Jose, CA) interfaced with a TriVersa NanoMate nanoelectrospray ion source (Advion BioSciences, Ithaca, NY). An Ultimate ^Plus^ nano-HPLC system with a Famous autosampler (Dionex Corporation, Sunnyvale, CA), was coupled with the TriVersa NanoMate. Peptides were loaded on a C18 µ-Precolumn™ Cartridge (5 µm, 100Å, 300 µm i.d.′ 5 mm) from the autosampler with a 25 µl sample loop at a flow rate of 15 µl/min. After injection of sample, 20 mL, and washing for 20 minutes, the precolumn was switched in line with the analytical column, a C18 PepMap100, 3 µm, 100 Å, 75 µm i.d. ′ 150 mm (Dionex Corporation, Sunnyvale, CA). Mobile phase B (80% acetonitrile/water+0.1% formic acid) was increased from 2% to 55% over 70 minutes, held for 5 minutes, increased to 95% over 20 minutes and held at 95% B for 5 minutes. The flow rate used was 250 nL/min and mobile phase A consisted of 5% acetonitrile/water+0.1% formic acid. The four most intense ions having a charge state between +2 to +4, determined from an initial survey scan from 300–1800 *m/z*, were selected for zoom scan and MS/MS. MS/MS was performed using an isolation width of 2 *m/z*, normalized collision energy of 35%, a minimum signal intensity of 1000 counts, and the dynamic exclusion option enabled. Once a certain ion is selected twice for MS/MS in 30 sec, this ion is excluded from being selected again for MS/MS during the next period of 120 sec. Dta files were created from the raw LTQ mass spectrometer LC-MS/MS data. The created dta files were then merged using the merge script tool from Matrix Science (http://www.matrixscience.com). The subsequent combined merge file was used to search the database of compiled *T. gondii* proteins using the following parameters with MASCOT [24] (in-house): trypsin, 2 missed cleavages; variable modifications of carbamidomethylation (Cys), deamidation (Asn and Gln) and oxidation (Met); monoisotopic masses; peptide mass tolerance of 3.0 Da; product ion mass tolerance of 0.6 Da. Peptides with a 95% significance score were accepted only. Each identified protein sequence was categorized according to the highest ion score of all of it's assigned peptides; 76% of the proteins had a high ion score more than 60, while 24% of the proteins had a high ion score between 30 and 60.

### EST data analysis

The EST dataset was obtained by downloading the NCBI EST “others” database (ftp://ftp.ncbi.nih.gov/blast/db/). The FASTA definition lines were searched and the sequences were selected for the organism name “*Toxoplasma gondii*”. A custom *T. gondii* EST database was constructed for the resulting 129,736 sequences. A TBLASTN (with an e-value requirement of 10^−10^ and without low complexity filtering) search was performed for each protein sequence against the custom database of *T. gondii* EST sequences. For each query sequence, the EST alignments were filtered to include only the ESTs with a sequence identity of greater or equal to 90% and an alignment length of 30 or more residues.

## Supporting Information

Table S1 Sequence annotation (PFAM, Transmembrane domains, Signal peptides) for sequence of each prediction type.
(0.04 MB DOC)
