# Supplementary material for: Computational Analysis and Experimental Validation of Gene Predictions in Toxoplasma gondii
Source: PLoS One. 2008 Dec 9;3(12):e3899. doi: 10.1371/journal.pone.0003899 (PMC2587701; doi:10.1371/journal.pone.0003899)
Supplement: Table S1 — Sequence annotation (PFAM, Transmembrane domains, Signal peptides) for sequence of each prediction type. (0.04 MB DOC) [file pone.0003899.s001.doc]

**Suppl. Table 1**

| **Predicted Protein Types** | **TigrScan** | **TwinScan** | **Glimmer** | **Release4** | **NR** |
| --- | --- | --- | --- | --- | --- |
| **Sequences (% of dataset) annotated with** |  |  |  |  |  |
| PFAM domain | 36% | 43% | 62% | 44% | 69% |
| Transmembrane segment | 21% | 19% | 28% | 24% | 26% |
| Signal Peptide | 13% | 16% | 11% | 16% | 20% |
